# An optimized translating ribosome affinity purification protocol for low-abundance *Drosophila* tissues

**DOI:** 10.1016/j.isci.2026.115530

**Published:** 2026-03-30

**Authors:** Leonor Miller-Fleming, Wing Hei Au, Alexander J. Whitworth

**Affiliations:** 1 MRC Mitochondrial Biology Unit, University of Cambridge, Cambridge Biomedical Campus, Cambridge CB2 0XY, UK

**Keywords:** genetics, molecular biology, neuroscience

## Abstract

Localized protein translation enables spatially restricted cellular dynamics, particularly in neurons, where specific mRNAs are translated in axons and dendrites far from the cell body. Translating ribosome affinity purification (TRAP) has been used to study axonal translation in rodents and cell-type-specific translation in *Drosophila*, but existing protocols are not optimized for axons, where material is extremely limited. Here, we present a highly sensitive TRAP protocol for isolating ribosome-bound mRNAs from low-input samples, enabling recovery of axonal mRNAs from *Drosophila* larval and adult (leg) motor neurons. RNA-seq identified axonally translated transcripts, including mRNAs encoding ribosomal and mitochondrial proteins, similar to those reported in axons of other species, indicating conservation of axonal translation in *Drosophila*. This low-input method enables analysis of local translation with *Drosophila* genetics across developmental stages, genetic backgrounds, and disease models, and can be adapted for rare genotypes, other tissues and model systems requiring high sensitivity.

## Highlights

• Highly optimized protocol for TRAP for very limited input quantities
• We highlight a number of caveats with existing transgenic reagents
• Axonal TRAP of motor neurons shows abundant ribosome and mitochondrial proteins

## Graphical abstract

**Figure:**
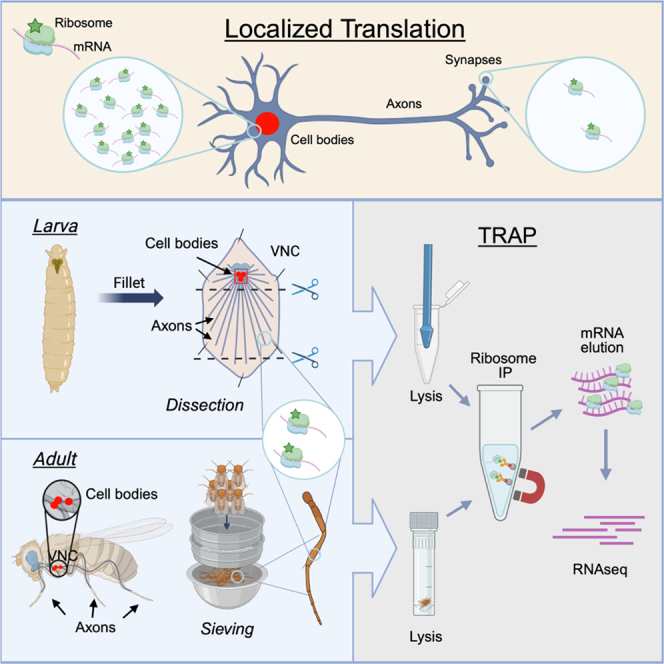

## Introduction

Regulated translation of localized mRNAs enables exquisite spatiotemporal control of gene expression in response to dynamic cellular needs. This is particularly critical in cells with elaborate architectures, such as neurons, where regions of high activity occur at great distances from the nucleus. Methods to interrogate subcellular pools of mRNAs (the transcriptome) and specifically those undergoing active translation (the translatome) have greatly advanced our understanding of the importance of localized translation.^1^ Consequently, local axonal translation has emerged as an important regulatory mechanism for neuronal function and long-term homeostasis.^1^^,^^2^^,^^3^ However, its role in the function and survival of mature neurons and synapses, and how dysregulation of this process could contribute to disease, is still poorly understood.^4^

The translating ribosome affinity purification (TRAP) method enables characterization of the translatome by expressing a tagged ribosomal protein in a specific tissue or cell-type, which becomes incorporated into mature ribosomes.^5^ The tagged ribosome-mRNA complexes are immunopurified and mRNAs are identified by RNA-seq. TRAP has been extensively used in mammalian systems, including the analysis of translatomes in neuronal subcellular compartments *in vivo* (e.g., rodent axons^6^^,^^7^), where ribosome abundance is very low (reviewed in the study by Garat et al.^8^). Although TRAP has been successfully applied to the analysis of *Drosophila* tissues *en masse*,^9^^,^^10^^,^^11^^,^^12^^,^^13^ we found that existing protocols were not sensitive enough for low-input samples, due to high background, poor ribosome binding, RNA degradation, and the requirement for large amounts of starting material to yield sufficient mRNA for sequencing.^9^^,^^10^^,^^12^^,^^13^ To overcome these limitations, we optimized the TRAP method for low-input tissues using a transgenic line expressing Flag-GFP-RpL10, previously used in TRAP experiments.^9^ After testing multiple protocol variations, we found that three factors were critical for successful TRAP: the choice of antibody, the timing of detergent addition to the lysate, and the time frame of the protocol to minimize RNA degradation. Here, we present a protocol optimized for low-abundance samples in *Drosophila,* which can be applied to the analysis of localized subcellular mRNA populations.

## Results

### Translating ribosome affinity purification optimization for limited material

Since robust and selective immunopurification of the tagged ribosome is central to the sensitivity and specificity of TRAP, we first optimized the IP conditions to minimize non-specific ribosome binding to the beads or antibody (see STAR Methods and Table S1 for details).

For initial protocol optimization, we used a UAS-Flag-GFP-RpL10 transgene, previously used for TRAP,^9^ expressed via OK371-GAL4—a widely used enhancer trap line residing in the *VGlut1* gene with robust expression in motor neurons (MNs).^14^ Expression driven by OK371-GAL4 would ensure that immunoprecipitation (IP) from whole-fly samples would yield sufficient material for testing the method while still being restricted to a specific cell type, i.e., a relatively small proportion of the sample. To control for non-specific binding, we used a transgenic line expressing soluble, cytosolic GFP.

**Figure 1:**
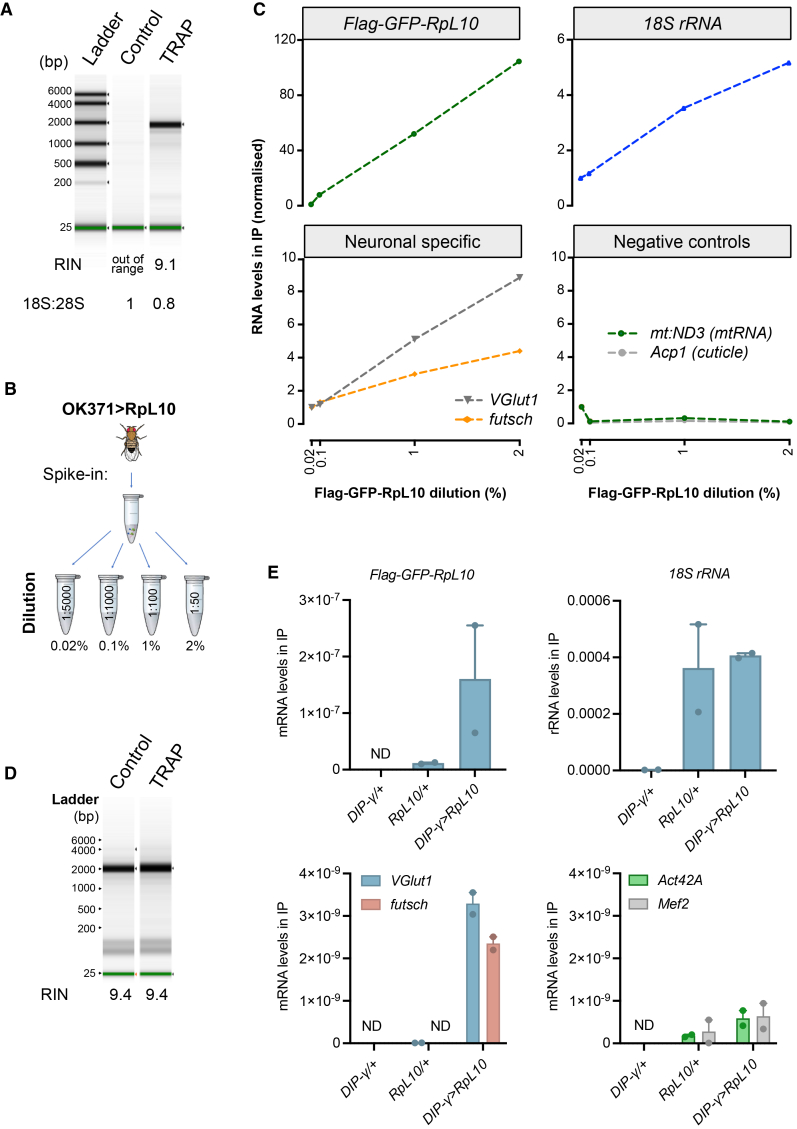
TRAP is a highly sensitive and specific technique to capture MN mRNAs (A) TapeStation electrophoresis of RNAs extracted from IPs carried out in whole flies expressing OK371>Flag-GFP-RpL10 (TRAP) or OK371>GFP (Control). *Drosophila 28S* rRNA is processed into 2 fragments that migrate similarly to the *18S* rRNA. The RIN value indicates the RNA integrity (RIN>8 indicates high-quality RNA). The ratio of *18S:28S* rRNA was calculated by RT-qPCR analysis. (B) Schematic illustrating the serial dilutions used to assess TRAP sensitivity. Lysates from flies expressing OK371>Flag-GFP-RpL10 were diluted with OK371-GAL4/+ lysates to final concentrations of 0.02, 0.1, 1, and 2% equivalent. (C) RT-qPCR analysis of indicated targets (*GFP*, *Vglut1*, *Futsch*, *18S* rRNA, *mt:ND3*, and *Acp1*) following ribosome IP from B shows the levels of RNAs extracted from the IP, normalized to the lowest OK371>Flag-GFP-RpL10 dilution (0.02%). (D) TapeStation electrophoresis of RNAs eluted from IPs from 10 thoraces of flies expressing DIP-γ>Flag-GFP-RpL10 (TRAP) or Flag-GFP-RpL10/+ (Control). (E) RT-qPCR analysis of DIP-γ>Flag-GFP-RpL10, as in D, against respective controls of GAL4 alone (DIP-γ/+) or UAS transgene alone (RpL10/+). Charts show mean ± SEM with biological replicates shown (*n* = 2). ND = not detected.

Specificity was confirmed by the presence of ribosomal RNA in the TRAP IP samples and absence in the GFP control, visualized by TapeStation electrophoresis of the RNAs eluted from beads (Figure 1A). Importantly, the levels of *18S* and *28S* rRNA extracted from the TRAP samples were comparable (Figure 1A), indicating that the isolated ribosomes were mostly intact.

To evaluate the TRAP sensitivity to low levels of labeled ribosomes and mRNA, we performed a dilution series using lysates from OK371>Flag-GFP-RpL10 flies mixed with lysates from the background genotype (OK371-GAL4/+) not expressing the transgene (Figure 1B). After extracting RNA from the immunopurified ribosomes, we quantified by real-time quantitative PCR (RT-qPCR) the *18S* rRNA, *Flag-GFP-RpL10* transgene (using primers against GFP), two neuronal mRNAs (*VGlut1* and *futsch)* and two non-specific transcripts, a mitochondrial mRNA which is translated by mitochondrial ribosomes (*mt:ND3)* and a cuticle-specific mRNA (*Acp1*) (Figure 1C). Notably, we detected the *Flag-GFP-RpL10* mRNA even in the most diluted TRAP sample (1:5000 or 0.02% equivalent of OK371>Flag-GFP-RpL10 lysate). Furthermore, the *18S* rRNA and neuron-specific transcripts correlated with the amount of diluted OK371>Flag-GFP-RpL10 lysate, while negative control transcripts did not. These results indicate that the TRAP signal is highly specific and sensitive, even when the number of labeled ribosomes is extremely low.

We next sought to further optimize the protocol to extract mRNAs from very limited cell populations. For this, we turned to DIP-γ-GAL4, which expresses in a much more restricted subset of MNs than OK371-GAL4.^15^^,^^16^^,^^17^ We unexpectedly observed substantial leaky expression from the UAS-Flag-GFP-RpL10 transgene in the absence of GAL4 induction (labeled as “control” on Figure 1D and “RpL10/+” in Figure S1A). We tested two other similar transgenes (two insertions of UAS-RpL3-3xFlag) and observed similar leaky expression (Figure S1B). Nonetheless, expression of Flag-GFP-RpL10 under the control of DIP-γ-GAL4 resulted in clear enrichment of the neuronal transcripts *VGlut1* and *futsch* in DIP-γ>Flag-GFP-RpL10 TRAP samples compared to the UAS-Flag-GFP-RpL10/+ controls (Figure 1E). Thus, despite the leaky expression, the UAS-Flag-GFP-RpL10 transgene is suitable for developing the TRAP protocol when using the appropriate non-driven (outcrossed) transgene as a control.

**Figure 2:**
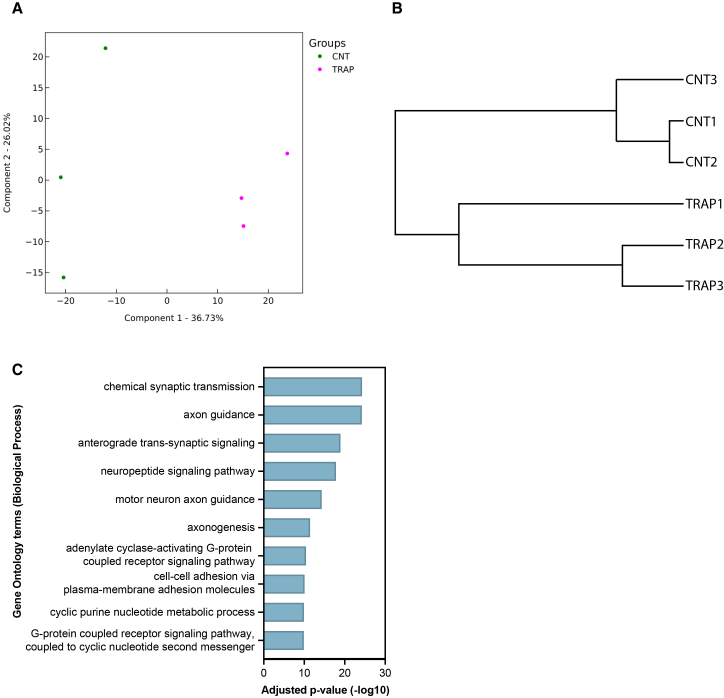
Optimized TRAP enables mRNA profiling from a restricted neuronal population in a small number of adult heads (A) Principal component analysis and (B) hierarchical clustering of TRAP RNA-seq data from 2-day-old adult heads expressing Flag-GFP-RpL10 in DIP-γ neurons (TRAP) and controls Flag-GFP-RpL10/+ (CNT). (C) Gene Ontology (GO) enrichment analysis (biological process terms) of transcripts enriched in DIP-γ-expressing neurons.

To further validate the TRAP method, we performed TRAP on 5 fly heads from DIP-γ>Flag-GFP-RpL10 or Flag-GFP-RpL10/+ genotypes. Translating mRNAs were then identified and quantified by RNA-seq. Principal-component analysis and hierarchical clustering of the RNA-seq outputs indicated that replicates were well separated into their respective biological groups (Figures 2A and 2B). The identification of 1974 differentially expressed genes (DEGs) in the DIP-γ>Flag-GFP-RpL10 samples showed a remarkable enrichment of neuronal genes (Table S2). Gene ontology (GO) analysis showed strong enrichment in neuronal-related terms (Figure 2C), such as synaptic transmission and axon guidance. Thus, these results demonstrate that the optimized TRAP protocol works successfully on a limited amount of material with very restricted expression.

### Translating ribosome affinity purification optimization for *Drosophila* axons

Next, we applied this protocol to identify mRNAs undergoing translation in MN axons in both larvae and adults, which, to our knowledge, has not been characterized in *Drosophila*.

**Figure 3:**
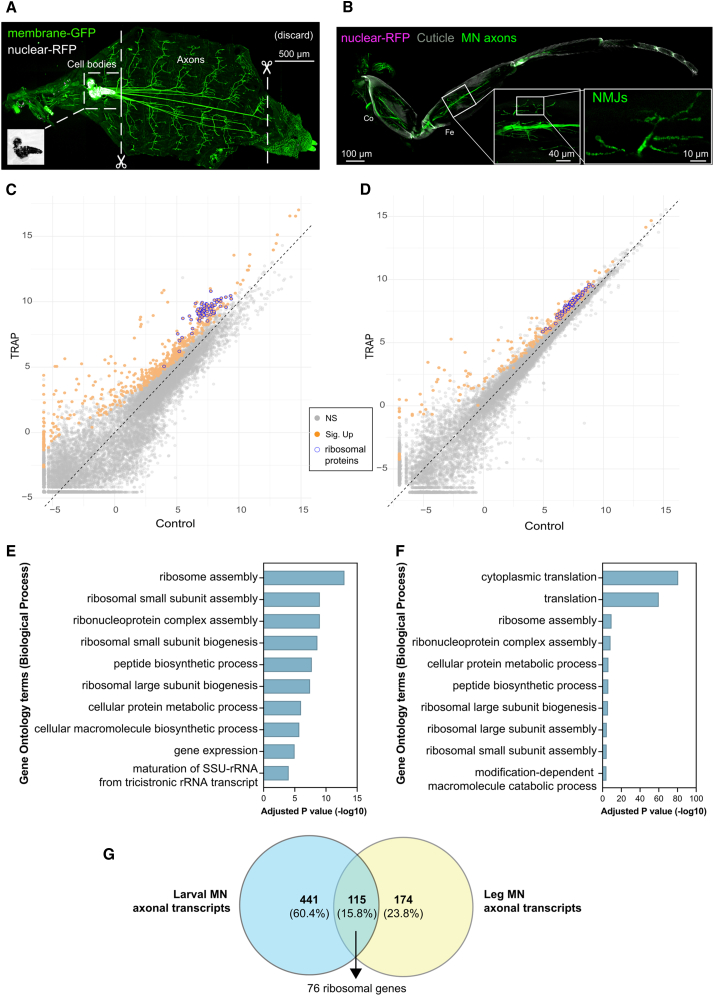
Application of optimized axonal TRAP in larva and adult leg MNs (A) Separating axons from soma in larvae. Confocal microscopy of an L3 larva showing membrane-GFP and nuclear-RFP expression in MNs driven by OK371-GAL4. Dashed lines represent the approximate dissection sites to separate axons from cell bodies. The anterior and posterior ends were discarded. Scale bars, 500 μm. (B) Expression pattern of DIP-γ-GAL4 in the leg labeled by expression of a membrane-GFP and nuclear-RFP. Axons of MNs are present in a large bundle in the coxa (Co), femur (Fe) and tibia. Expression of nuclear-RFP is not observed in the leg as the cell bodies are located in the thoracic ventral nerve cord. (C and D) Scatterplots comparing transcript levels between TRAP versus control genotypes in larval (C) and adult leg samples (D) expressed as log_2_-transformed counts per million mapped reads (log_2_ CPM). (E and F) Gene ontology (GO) enrichment analysis of TRAP-enriched genes from (E) larval and (F) leg samples. Top biological process terms are shown, ranked by adjusted *p* value. (G) Venn diagram showing the overlap between transcripts significantly enriched in TRAP datasets from larval and leg samples (adjusted *p* < 0.05).

The larval neuromuscular system is extremely well studied. Their anatomical arrangement means that MN cell bodies (soma), located in the ventral ganglion, are spatially separated from their distal axons and synapses, allowing their physical isolation by simple dissection (Figures 3A and S2A). Decades of work have yielded robust genetic tools for cell-type-specific expression. After testing several MN GAL4 driver lines, we confirmed that OK371-GAL4 is indeed highly restricted to MNs (Figure 3A). We therefore performed TRAP from larval axonal sections expressing OK371>Flag-GFP-RpL10 to isolate ribosome-bound mRNAs from larval MN axons (Figures S3A–S3C).

Similarly, adult leg MNs have their cell bodies in the thoracic ventral nerve cord and project into the leg to innervate the muscles, providing a simple setup to physically separate axons from cell bodies (Figure S2B). Unfortunately, OK371-GAL4 labeled a few sensory neurons in the leg (Figure S4A) and so was not suitable for leg axon-TRAP. We found that the DIP-γ-GAL4 has an MN axon-specific expression pattern in the leg, by detecting no nuclear-RFP signal and no obvious membrane-GFP labeled cell body-like structures (Figure 3B), consistent with a previous report.^16^ Importantly, expression of Flag-GFP-RpL10 did not affect motor function, as shown by no effects on climbing ability over 20 days (Figure S4B). Thus, to specifically identify MN axonal transcripts in adult legs, we used DIP-γ-GAL4 to drive Flag-GFP-RpL10 expression (Figure S3D) and physically isolate axon-localized ribosomes by simple isolation of legs by snap-freezing 2-day-old adults, vortexing and subsequent separation of legs from bodies by sieving (Figure S2B).

Following axon-TRAP of both larval and adult MNs, eluted RNAs were tested for their integrity by electrophoresis using a TapeStation, and all samples were consistently of high quality (RNA integrity number (RIN) > 8) (Figures S5A and S5B). The samples were then subjected to RNA-seq analysis. Gene body coverage was assessed across normalized transcript lengths and demonstrated read distribution without significant 3′ end bias, particularly in the leg samples, indicating preserved RNA integrity and even sequencing across transcript length (Figures S5C and S5D).

RNA-seq analysis revealed 556 transcripts in the larval axon-TRAP (Figure 3C; Table S3) and 289 in the leg MN axon-TRAP (Figure 3D; Table S4), significantly enriched relative to their respective Flag-GFP-RpL10/+ controls. Among the identified transcripts in both datasets, we observed a striking number of ribosomal protein mRNAs: 81 in larval axons (Figures 3C and 3E; Table S3) and 78 in adult leg MN axons (Figures 3D and 3F; Table S4), accounting for 27% (78/289) of all transcripts in the leg TRAP. Notably, 115 transcripts were shared between the larval and leg TRAP datasets, of which 76 were ribosomal protein mRNAs (Figure 3G). Ribosomal protein mRNAs are known to be localized in axons and undergo local translation in different neuron types in other model organisms,^18^^,^^19^^,^^20^^,^^21^^,^^22^ supporting a conserved mechanism of axonal protein synthesis. In addition to ribosomal proteins, the shared transcripts included mitochondrial components, such as *Tom5* and *Tom7*. Interestingly, it also included the transcript encoding translationally controlled tumor protein Tctp, which was previously reported to be locally translated in *Xenopus laevis* axons, playing a role in axonal mitochondrial homeostasis and axon development.^23^^,^^24^ We also found a number of neuronal transcripts of significant interest (Table S3), including *caz* in the larval axon-TRAP, the homologue of FUS, an RNA-binding protein known to be involved in RNA transport and local translation in neurons, and mutated in familial forms of motor neuron disease.^25^^,^^26^

The presence of these well-established axonally translated mRNAs in our dataset supports the validity of our method for capturing axonal translation in *Drosophila*. Nonetheless, further validation is required to confirm their local translation and functional relevance in *Drosophila* MN axons.

## Discussion

The TRAP protocol has emerged as a powerful approach to selectively identify cell-type-specific translating mRNAs, preserving the physiological context, without the need for gross tissue dissociation. This is particularly important for neurons, where subcellular compartmentalization of translation plays a critical role in neuronal development, plasticity, and function.^1^^,^^2^^,^^3^ Notably, TRAP has been successfully employed to study axonal translation *in vivo* in rodents,^6^^,^^7^ providing important insights into local protein synthesis. The quantity of ribosomes in axons is very low,^8^ requiring TRAP to be highly sensitive. The ability to apply TRAP to *Drosophila* would allow the exploitation of several advantages of this model—its powerful genetic toolkit, short generation time and suitability for large-scale screening—allowing the study of axonal translation in a larger scale and to address fundamental and disease-relevant questions that are more challenging in vertebrate models. Here, we present a highly sensitive TRAP protocol optimized for the identification of actively translating mRNAs of low-abundant *Drosophila* tissues, including mRNAs from larval and adult leg MNs axons.

To effectively use the TRAP protocol for low-abundance samples, where labeled ribosomes are scarce, we increased the sensitivity and specificity of the TRAP protocol. A major challenge of TRAP, as with any RNA IP protocol, is avoiding non-specific ribosome or mRNA binding to the beads and/or antibodies. For TRAP, an additional critical consideration that can make specificity harder to achieve is ensuring that the immunopurified ribosomes remain intact and associated to the mRNAs being translated. We found that the choice of antibody-bead combination, as well as the timing of detergent addition to the samples, was crucial for reducing non-specific binding (Table S1). By increasing the IP specificity, we were able to improve its sensitivity and reduce the required input material. Minimizing incubation times was also an important step to ensure ribosome and RNA integrity, further contributing to the increased TRAP sensitivity (Table S1).

Importantly, we found leaky expression of the UAS-Flag-GFP-RpL10 and UAS-RpL3-3xFlag transgenes in the absence of GAL4, which is a known phenomenon for certain UAS transgenes. Although this initially raised concerns about specificity, we were able to mitigate background signal by using the appropriate non-driven (outcrossed) transgene. This allowed us to reliably detect neuronal-enriched transcripts, even with restricted GAL4 drivers. These findings highlight the importance of including appropriate genetic controls when using UAS-based TRAP lines, particularly in low-expression contexts. This finding also highlights careful interpretation of published TRAP datasets using similar transgenes, as background expression can compromise cell-type specificity.

We also show the successful use of TRAP in identifying ribosome-bound mRNAs from both *Drosophila* larval and adult MN axons for the first time. We found ribosomal protein mRNAs were enriched in both larval and adult MN axonal datasets, whereas there was no enrichment in the whole-head dataset, consistent with previous reports in mammalian systems.^18^^,^^19^^,^^20^^,^^21^^,^^22^ The translation of ribosomal proteins in axons has been suggested to be required for supplying additional ribosomal proteins to maintain and/or modify local ribosomal function far from the nucleolus, which is where ribosomes are assembled.^19^^,^^27^ The presence of mitochondrial components and axonally translated transcripts such as *Tctp* and *caz*, the homologue of the human ALS-associated gene *FUS*, further supports the specificity and functional relevance of the captured transcripts and suggests conservation of local translation across species. Interestingly, the populations of ribosome-associated mRNAs from larval and adult MNs showed little overlap indicating that different cell-biological processes predominate between these stages. Another important consideration in the context of the TRAP methodology would be to discriminate between actively translating ribosomes and ribosome-associated or stalled complexes by performing ribosome run-off using compounds such as harringtonine.^6^

In summary, our optimized TRAP protocol enables sensitive and specific profiling of ribosome-bound mRNAs from rare cell types, anatomically restricted neuronal populations, and genetically challenging *Drosophila* backgrounds. This protocol will allow the study of local translation in combination with powerful *Drosophila* genetic tools, enabling detailed investigation of axonal translation across developmental stages, genetic conditions, and disease models.

### Limitations of the study

Our protocol achieves higher sensitivity than other published *Drosophila* protocols, allowing us to investigate the translatome of low-input samples and axons, however, there are still some limitations. Although using the non-driven UAS-Flag-TEV-GFP-RpL10 transgene as a control seemed to solve the leaky expression problem (shown by a highly enriched neuronal dataset in the head TRAP), we cannot exclude that some low-abundant transcripts were not detected due to antibody binding competition with other abundant mRNAs that came from the leaky expression.

Despite the increased sensitivity compared with other protocols, it is not sensitive enough to be used for individual cells. However, although single-cell RNA-seq is revolutionizing cell-type-specific transcriptomics and Ribo-seq is being developed to assess translation at the level of individual cells,^28^ these techniques remain technically limited in spatial resolution.

Although we present high-confidence axonal translatomes for larval and adult leg MN axons, further validation will be necessary. This includes independent validation of the axonal localization of transcripts of interest using complementary approaches such as single-molecule fluorescence *in situ* hybridization (smFISH) as well as other strategies to confirm their active translation state and define their functional roles. Nevertheless, the identification of axonal ribosome-associated transcripts in both developmental stages opens up new avenues for investigating how local translation contributes to MN function, maintenance, and how dysregulation of their translation can contribute for neurodegeneration.

## Resource availability

### Lead contact

Dr. Alexander J. Whitworth (ajw69@cam.ac.uk).

### Materials availability

This study did not generate new or unique reagents.

### Data and code availability

• RNA-seq datasets have been deposited at GEO (GEO: GSE313171).
• This paper does not report original code and data analysis methods are described in the STAR Methods.
• Any additional information required to re-analyse the data reported in this study is available from the lead contact upon request.

## Acknowledgments

This work was supported by European Research Council Starting grant (309742), 10.13039/501100000265Medical Research Council core funding (MC_UU_0028/6), Medical Research Council project grants (MR/V003933/1), and 10.13039/501100000406Motor Neurone Disease Association grant (Whitworth/Apr17/857-79). We thank Madeleine Twyning, Alvaro Sanchez-Martinez, Victoria Hewitt, and Pedro Rebelo-Guiomar (MRC-MBU, University of Cambridge) for help with dissections and Simon Andrews (Babraham Institute) for guidance in RNA-seq analysis. We thank Robert Carrillo and Lawrence Zipursky for kindly providing fly stocks. Schematic drawings in the Graphical Abstract and Figures were created in BioRender.

## Author contributions

Conceptualization, methodology, formal analysis, investigation, writing – original draft, writing – review and editing, visualization, supervision, funding acquisition, L.M.-F.; conceptualization, methodology, investigation, writing – review and editing, W.H.A.; conceptualization, validation, formal analysis, writing – original draft, writing – review and editing, supervision, project administration, funding acquisition, A.J.W.

## Declaration of interests

The authors declare no competing interests.

## STAR★Methods

### Key resources table

**Table:** 

REAGENT or RESOURCE	SOURCE	IDENTIFIER
**Antibodies**		

Monoclonal mouse anti-FLAG M2 antibody	Sigma-Aldrich	Cat# F1804; RRID: AB_262044
Secondary antibody HRP-conjugated goat anti-mouse IgG H&L	Abcam	Cat# ab6789; RRID: AB_95543

**Chemicals, peptides, and recombinant proteins**		

Cycloheximide	Sigma-Aldrich	Cat# C7698
DNase I, RNase-free	Thermofisher scientific	Cat# AM2222
Dynabeads™ Protein G for immunoprecipitation-1 mL	Thermofisher scientific	Cat# 10003D
HEPES buffer, 1M Solution, pH 7.3	Fisher BioReagents	Cat# BP299-100
KCl (2 M), RNase-free	Invitrogen	Cat# AM9640G
MgCl_2_ (1 M), RNase free	Invitrogen	Cat# AM9530G
Nonidet P-40 substitute (NP-40)	VWR	Cat# E109
Nuclease-free water (not DEPC-Treated)	Invitrogen	Cat# 10526945
Phosphate-Buffered Saline (PBS) (10X) pH 7.4, RNase-free	Thermo Fisher Scientific	Cat# AM9624
RNaseOUT	Invitrogen	Cat# 10777019
TRI reagent	Sigma-Aldrich	Cat# T3934
Tween 20, molecular biology grade	Merck	Cat# 655204

**Critical commercial assays**		

High Sensitivity RNA ScreenTape	Agilent	Cat# 5067-5579
High Sensitivity RNA ScreenTape Sample Buffer	Agilent	Cat# 5067-5580
High Sensitivity RNA ScreenTape Ladder	Agilent	Cat# 5067-5581
TapeStation loading tips	Agilent	Cat# 5067-5599
Maxima H Minus cDNA Synthesis Kit with dsDNase	Thermo Fisher Scientific	Cat# M1681
PowerUp™ SYBR™ Green Master Mix for qPCR	Thermo Fisher Scientific	Cat# A25741

**Deposited data**		

RNA-seq datasets	GEO (Gene Expression Ominbus)	GSE313171

**Experimental models: Organisms/strains**		

*D. melanogaster*: DIP-γ-GAL4 (MI03222-GAL4)	Bloomington *Drosophila* Stock Center	RRID: BDSC_90315
*D. melanogaster*: UAS-Flag-TEV-GFP-RpL10	Lawrence Zipursky lab	RRID: FlyBase_FBal0319196
*D. melanogaster*: OK371-GAL4	Bloomington *Drosophila* Stock Center	RRID: BDSC_26160
*D. melanogaster*: UAS-mCD8-GFP	Bloomington *Drosophila* Stock Center	RRID: BDSC_5137
*D. melanogaster*: UAS-NLS-RFP	Bloomington *Drosophila* Stock Center	RRID: BDSC_8547
*D. melanogaster*: UAS-GFP	Bloomington *Drosophila* Stock Center	RRID: BDSC_1522
*D. melanogaster*: DIP-γ-GAL4 (MI03222-GAL4)	Bloomington *Drosophila* Stock Center	RRID: BDSC_90315
*D. melanogaster*: UAS-Flag-TEV-GFP-RpL10	Lawrence Zipursky lab	RRID: FlyBase_FBal0319196
*D. melanogaster*: OK371-GAL4	Bloomington *Drosophila* Stock Center	RRID: BDSC_26160
*D. melanogaster*: UAS-mCD8-GFP	Bloomington *Drosophila* Stock Center	RRID: BDSC_5137
*D. melanogaster*: UAS-NLS-RFP	Bloomington *Drosophila* Stock Center	RRID: BDSC_8547
*D. melanogaster*: UAS-GFP	Bloomington *Drosophila* Stock Center	RRID: BDSC_1522

**Oligonucleotides**		

See Table S1 for primers used in this study.	This study	

**Software and algorithms**		

SAMtools	Li et al.^29^	http://samtools.sourceforge.net/
GraphPad Prism	GraphPad Prism	https://www.graphpad.com/
BioRender	BioRender	https://www.biorender.com/
Trim Galore	Babraham boinformatics group	https://www.bioinformatics.babraham.ac.uk/projects/trim_galore/
FASTQC	Babraham boinformatics group	www.bioinformatics.babraham.ac.uk/projects/fastqc/
HISAT2	Kim et al.^30^	https://daehwankimlab.github.io/hisat2/
DESeq2	Love et al.^31^	https://bioconductor.org/packages/release/bioc/html/DESeq2.html
MultiQC	Ewels et al.^32^	https://github.com/MultiQC/MultiQC
R Studio	Posit team	https://posit.co/download/rstudio-desktop/
Seqmonk	Babraham boinformatics group	https://www.bioinformatics.babraham.ac.uk/projects/seqmonk/
RSeQC	Wang et al.^33^	https://rseqc.sourceforge.net/

**Other**		

RNase-free disposable pellet pestles	Fisher Scientific	Cat# 13236679
Magnetic Separation Rack	New England Biolabs	Cat# S1509S
Minilys personal homogeniser	Bertin Technologies	Cat# P000673-MLYS0-A
Vannas spring scissors	Fine Science Tools	Cat# 15000-00
Razor blades	Fisher Scientific	Cat# Azpack™ 11904325
Dumont #5 - Fine Forceps	Fine Bioscience tools	Cat# 11254-20

### Experimental model and study participant details

*Drosophila melanogaster* flies were maintained under standard conditions at 25 °C with 65 % relative humidity in a temperature-controlled incubator on a 12 h:12 h light:dark cycle. Flies were reared on standard cornmeal-based food containing malt extract, molasses, cornmeal, yeast, agar, soya powder, water, propionic acid, and nipagin. The sex and age of animals used is detailed in individual method sections. DIP-γ-GAL4 (MI03222-GAL4; BDSC:90315)^15^^,^^16^^,^^17^ and UAS-Flag-TEV-GFP-RpL10^9^ were kindly provided by Robert Carrillo and Lawrence Zipursky, respectively. OK371-GAL4 (BDSC:26160), UAS-mCD8-GFP (BDSC:5137), UAS-NLS-RFP (BDSC:8547) and UAS-GFP (BDSC:1522) lines were obtained from the Bloomington *Drosophila* Stock Center. No ethical approval was needed for this study. Governance of the use of genetically modified organisms is reviewed by local committee and approved under document RA-GMO-Whitworth-2015-1.

### Method details

#### Climbing assay

The repetitive iteration startle-induced negative geotaxis (RISING or “climbing”) assay was performed using a counter-current apparatus as previously described.^34^ Briefly, groups of approximately 20 male flies were taken into a temperature-controlled room and allowed to acclimatise for 30 minutes and then transferred to the assay tubes for another 30 minutes. Flies were placed into the first chamber of the apparatus, tapped to the bottom, and given 10 seconds to climb a distance of 10 cm. Flies that climbed above 10 cm were moved into the next tube. This procedure was repeated five times, and the number of flies that remained in each chamber was counted. An average score was calculated and expressed as a climbing index. For climbing assays of the aged flies, the flies were transferred to fresh tubes containing food every 2–3 days.

#### Immunoblotting

The abdomen of two (2 day-old) male flies per sample was excised and discarded and the remaining tissues (thoraces, heads and legs) were lysed in RIPA lysis buffer (50 mM pH 7.4 Tris, 1 M NaCl, 0.1 % SDS, 0.5 % sodium deoxycholate, 1 % NP-40; cOmplete mini EDTA-free protease inhibitors (Roche; 4693159001)) and diluted with Laemmli Sample Buffer (Bio-Rad; 1610747). Lysates were incubated at 95 °C for 10 minutes and then centrifuged at 10,000 rpm for 5 minutes to remove debris. Lysates were equally loaded and separated by SDS-PAGE in 4–20 % Mini-PROTEAN® TGX™ Precast Protein Gels (Bio-Rad; 4561096) and transferred onto a nitrocellulose membrane (Bio-Rad; 1704158) via Trans-Blot Turbo Transfer System (Bio-Rad). Membranes were blocked for 1 hour at room temperature with 5 % (wt/vol) dried skimmed milk powder (Marvel Instant Milk) in TBS containing 0.1 % Tween-20 (TBS-T) and incubated overnight at 4 °C with an α-GFP (1:1000; Abcam; ab1218) or an α-Flag antibody (1:1000; Sigma-Aldrich; F1804). Incubation with the secondary antibody HRP-conjugated goat anti-mouse IgG H&L (1:5000; Abcam; ab6789) diluted in 5 % milk in TBS-T was for 1 hour at room temperature.

#### Microscopy

L3 wandering larvae were selected and dissected as fillets on Sylgard™ plates as previously described.^35^^,^^36^ Samples were fixed in 4 % paraformaldehyde in PBS for 20 min and washed three times with PBS. Legs were carefully cut using fine scissors and mounted immediately in VectaShield antifade mounting medium. Images were acquired using a Zeiss LSM880 confocal microscope (Carl Zeiss MicroImaging). Whole legs and larvae were imaged using a Plan-Apochromat 20×/0.8 NA air objective in tile scan mode. Neuromuscular junctions were also imaged using the same 20× objective. Cell bodies were imaged using a Plan-Apochromat 63×/1.40 NA oil-immersion objective, while axons were imaged using an EC Plan-Neofluar 40×/1.30 NA oil-immersion objective (DIC M27). Image analysis was performed using FIJI.

#### Translating Ribosome Affinity Purification (TRAP)

##### Fly rearing and tissue harvesting

For TRAP experiments, virgin females (2–5 days old) carrying UAS-Flag-GFP-RpL10 were crossed either to DIP-γ-GAL4 males for adult TRAP or OK371-GAL4 males for larval TRAP. Control samples were generated by crossing UAS-Flag-GFP-RpL10 females to w^1118^ males and selecting UAS-Flag-GFP-RpL10/+ males and females for analysis. For initial tests, GFP-only controls were used by crossing UAS-GFP virgin females to either DIP-γ-GAL4 or OK371-GAL4 males. Crosses were set up at consistent ratios of 7 females to 3 males in vials, and 10 females to 4 males in bottles. Crosses were flipped every two days to maintain healthy progeny.

For adult TRAP, DIP-γ-GAL4/UAS-Flag-GFP-RpL10 and UAS-Flag-GFP-RpL10/+ flies were collected shortly after eclosion and aged for 2 days at 25 °C. For head TRAP, five males per biological replicate were briefly anesthetised with CO_2_, flash-frozen in liquid nitrogen, and stored at −70 °C. For leg axon-TRAP, 250 flies (male and female, 1:1 ratio) were collected per biological replicate, in small batches under CO_2_ (to avoid overexposure). Heads were quickly removed with a razor blade, and the remaining bodies were then flash-frozen and stored at −70 °C.

Note: We recommend collecting flies of the exact same age and not older than 2 days, as we observed that RNA levels decline drastically after the first few days of adulthood, as previously reported.^37^

For larval axon-TRAP, 30 wandering third instar (L3) larvae were selected for each biological replicate with the genotypes: OK371>UAS-Flag-GFP-RpL10 and UAS-Flag-GFP-RpL10/+. Larvae were rapidly dissected as fillets on Sylgard™ plates in Schneider’s medium as previously described.^35^^,^^36^ Salivary glands and all other internal organs were carefully removed with forceps, preserving the VNC and its associated axons. An axonal section was then isolated by using spring scissors to cut out and discard both the anterior (including the VNC) and posterior ends (just before the spiracles). A view of the fillet is shown in Figures 3A and S2A, with the cut lines indicated by dotted lines. Axonal sections were immediately transferred into DNA LoBind Tubes (Eppendorf; 0030108051), kept on dry ice (to collect up to 10 sections per tube) and later stored at −70 °C.

Three biological replicates were performed per condition for all TRAP experiments unless otherwise stated.

Note: Larval dissections should be practised beforehand to prevent damage to the CNS.

Dissection pins may be left with sections in the tubes, as they do not interfere with lysis.

##### Preparation of buffers

Buffers were prepared the day before the TRAP experiment, except for the addition of cycloheximide (CHX), protease and RNase inhibitors and DTT, which were added fresh on the day of the experiment just before starting the lysis. Polysome lysis buffer (PLB) was made up of 20 mM HEPES pH 7.3, 150 mM KCl, 5 mM MgCl_2_, 1 mM DTT, RNaseOUT 400 U/mL, 100 μg/mL cycloheximide (CHX), protease inhibitors) and wash buffer 20 mM HEPES pH 7.3, 350 mM KCl, 5 mM MgCl_2_, 1 % (v/v) NP-40, 1 mM DTT, 100 μg/mL CHX. Buffers were stored at 4°C or on ice during the experiment.

Note: We recommend to clean pipettes and surfaces with RNaseZAP (Sigma-Aldrich; R2020) and to use disposable spatulas (Merck; Z561762) to weigh chemical compounds to prevent RNase contamination. Although adding a detergent to the lysis buffer is optimal for lysis efficiency, we do not recommend including it, particularly for larval tissues, as it substantially increases the non-specific binding of RNAs to the beads. The best outcome is when NP-40 is added only after lysis as described below.

##### Pre-clearing beads and bead-antibody conjugation

Conjugation of beads with antibody was performed the day before the TRAP experiment. For this, 15 μL of Dynabeads per sample were collected and resuspended in 500 μL of PBS containing 0.02 % Tween-20 (PBS-T). To wash the beads, the tube containing the beads was placed on a magnetic rack, the beads were allowed to attach to the side of the wall containing the magnet, the supernatant was removed and quickly resuspended in 500 μL of PBS-T to prevent beads from drying. This step was repeated twice. Finally, the beads were resuspended in 500 μL of PBS-T containing 1.5 μL (1.5 μg) of anti-Flag antibody per sample and the mixture was incubated overnight at 4 °C. The following day, conjugated beads were washed three times with polysome buffer (without RNase/protease inhibitors, cycloheximide, and DTT, but with 0.5 % NP-40), aliquoted into the required number of 1.5 mL DNA LoBind tubes and stored in the same buffer until use.

Beads for pre-clearing (20 μL of Dynabeads per sample) were also equilibrated the day before the experiment. They were washed three times with polysome buffer (without RNase/protease inhibitors, cycloheximide, and DTT, but with 0.5 % NP-40), as described above, also aliquoted into the required number of 1.5 mL DNA LoBind tubes and stored in the same buffer until use.

Note: The type of beads and antibody are crucial for the IP success, not only in terms of specificity but also sensitivity. We also tested the ChromoTek GFP-Trap Magnetic Agarose beads and although they were very efficient for ribosomal pull down, we lost the specificity of the IP (verified by RT-qPCR). We tested other antibodies, including two anti-GFP: Abcam ab290 and DSHB NeuroMab 86/8, which substantially increased the RNA background in the control samples.

The buffer used to wash and equilibrate the beads requires detergent, as the beads do not attach well to the side of the tube and are lost easily if no detergent is used.

##### Tissue lysis

Tissue lysis was carried out on the day of the experiment in a cold room to prevent RNA degradation. The lysis procedure was initiated only after all buffers were prepared and ice-cold, all tubes were labelled, all materials (including pipette tips, tubes, microcentrifuge) were cooled to 4 °C, Dynabeads were conjugated with antibody, and pre-cleared Dynabeads equilibrated.

For collection of heads, frozen flies were separated using a series of 5 cm diameter stainless steel sieves (400 and 710 μm pore sizes) and a stainless-steel collection bowl kept on dry ice. Heads were collected from the 400 μm mesh top sieve and immediately transferred to 2 mL screw-cap tubes (Precellys; P000945-LysKO-A.O) using a dry-ice-cooled funnel.

For collection of legs, frozen animals (without heads) were separated using the same setup as for heads, collecting the legs from an aluminium dish placed at the bottom of the sieves directly into the 2 mL screw-cap tubes using a dry-ice-cooled funnel.

Note: All material that comes into contact with the frozen animals has to be kept dry-ice cold. Animals must remain frozen at all times to maintain RNA integrity.

For head and leg tissue lysis, 2 mm zirconium oxide beads (Next Advance; ZROB20-RNA) were aliquoted into tubes (corresponding to a volume of approximately 150 μL) and cooled on ice. Beads were then transferred to the 2 mL screw-cap tubes containing the frozen tissues. Next, 1 mL of ice-cold PLB was added, and tissues were homogenised using a Minilys personal homogeniser (Bertin Technologies) at 5,000 rpm for 10 seconds, twice, with a 5-minute interval on ice between runs.

Note: It is important to note that the order in which legs, beads and buffer are added to the 2 mL screw-cap tubes should not be changed. Since the lysis buffer lacks detergent, adding the legs after the buffer can result in extremely poor lysis, as the legs tend to stick to the tube walls.

For larval tissue lysis, ice-cold PLB was added at a ratio of 15 μL per axonal section. Tissues were lysed rapidly using an RNase-free pestle with approximately 15 strokes. After lysis, samples were briefly centrifuged in a mini centrifuge to collect all lysate at the bottom of the tube. Lysates corresponding to 30 sections were pooled, and 150 μL of additional PLB was added to the combined samples. Next, lysates were centrifuged at 2,000 x g at 4 °C for 2 minutes. Pellets containing debris were discarded, and the supernatants were carefully transferred to new 1.5 mL DNA LoBind tubes containing 10 % NP-40 to achieve a final concentration of 0.5 %. Samples were gently mixed by inverting the tubes and then incubated on ice for 5 minutes. They were then centrifuged at 20,000 x g for 12 minutes at 4 °C and the supernatants were transferred to new tubes.

##### Pre-clear

Equilibrated pre-clearing beads, previously stored in PLB, were placed in a magnetic rack, the buffer was removed, and immediately replaced with the prepared lysates. Samples were left on a rotator with gentle rotation for 30 minutes at 4 °C.

##### Immunoprecipitation

Antibody-conjugated beads were washed three times with PLB containing 0.5 % NP-40, without RNase and protease inhibitors, CHX, and DTT, using a magnetic rack. The washed beads were then aliquoted into new 1.5 mL DNA LoBind tubes, and the PLB was replaced with the pre-cleared lysate using a magnetic rack. Samples were incubated on a rotator for 2.5 hours at 4 °C. Following incubation, samples were briefly centrifuged in a mini centrifuge and placed on a magnetic rack. The supernatant (unbound fraction) was carefully collected from each sample and immediately replaced with 500 μL of wash buffer, one at a time, to prevent the beads from drying. The unbound lysate was stored at −70 °C for later analysis. Beads were then washed five times with wash buffer. After the final wash, the beads were resuspended in 100 μL of wash buffer containing 5 U of DNase I and incubated at 37 °C for 2 minutes. The samples were subsequently washed once more by adding 900 μL of wash buffer, placing them on the magnetic rack, removing the buffer and resuspending beads in 100 μL of wash buffer. Next, the resuspended beads were transferred into a new tube containing 300 μL of TRIzol LS. Samples were then stored at −70°C.

Note: Pre-clearing and IP times were optimised to have the maximum of ribosomal IP, the minimum RNA background binding, and to maintain RNA integrity. Working in a cold environment was also important for RNA integrity. Keeping the beads wet at all times is crucial for the IP success.

##### RNA extraction and RNA-Seq

RNA was extracted from IPs using Direct-zol RNA microprep kit (Zymo Research; R2060) following the manufacturer’s instructions. Elution was done using 10 μl of nuclease-free water. RNA integrity and concentration were assessed using 1 μL of the eluate on a TapeStation 2200 (High Sensitivity RNA ScreenTape reagents; Agilent), with all samples exhibiting a minimum RNA integrity number (RIN) of 8. From head IPs, 5 ng of RNA was eluted per sample, from larval axonal section IPs, 2.4 ng was extracted, and from leg IPs, 4 ng was obtained.

RNA was sequenced by an external provider, using the Smart-Seq HT KIT (Takara) to synthesise and amplify the cDNA. Libraries were sequenced on an Illumina platform (2 × 150 bp paired-end reads), generating approximately 20 million raw paired-end reads per sample.

##### RNA-Seq analysis

Trim Galore was used for adapter removal and quality trimming.^38^ FASTQC was used for data quality control assessment.^39^ Trimmed reads were aligned to the *Drosophila melanogaster* genome (BDGP6, Ensembl v95) using HISAT2 v2.2.1^30^ with splice sites extracted from the GTF annotation. Alignments were converted to BAM, sorted and indexed using SAMtools v1.12.^29^ RNA-seq quality control was performed with RSeqC^33^ and Seqmonk.^40^ Mapped reads were visualised and quantified using Seqmonk. Genes with no counts (defined as fewer than 1 count in at least one sample) were removed.

MultiQC was used to group results from the various bioinformatic analyses.^32^ Gene ontology was analysed using the web-based tool FlyEnrichr^41^ using the transcripts that were statistically significantly more abundant in the TRAP samples compared to control (FDR<0.05). Scatter plots were created with R studio using the packages ggplot2 and ggrepel.

One replicate of the control UAS-Flag-GFP-RpL10/+ leg axon-TRAP was excluded from analyses due to high DNA contamination.

#### Quantitative real-time PCR (RT-qPCR)

mRNA was reverse transcribed with the Maxima H Minus cDNA Synthesis Kit with dsDNase (Thermo Fisher Scientific; M1681) following the manufacturer’s instructions. RT-qPCR was performed using PowerUp™ SYBR™ Green Master Mix for qPCR (Thermo Fisher Scientific; A25741) following manufacturer’s instructions, in a QuantStudio™ 3 Real-Time PCR System (Applied Biosystems). The primers used are described Table S5. To distinguish endogenous RpL10 from the transgene, we used primers that amplify GFP.

### Quantification and statistical analysis

Climbing data were analysed using the Kruskal–Wallis non-parametric test with Dunn’s correction for multiple comparisons using GraphPad Prism 9 (SCR_002798). RNAseq gene expression analysis was performed using the Seqmonk built-in statistical tool DESeq2.^31^ RT-qPCR relative quantification was performed using the comparative CT method, taking into account the PCR primer efficiency. Primer efficiency (e) was calculated using a standard curve with 5 cDNA serial dilutions. CTs were transformed into quantity, using the equation e^-CT^.^42^ Plots were generated in GraphPad Prism 9 (SCR_002798).

## Supplemental information

Document S1. Figures S1–S5 and Tables S1 and S5

Table S2. List of significantly differentially expressed genes identified by DESeq2 in DIP-γ labeled neurons from adult heads

Table S3. List of significantly differentially expressed genes identified by DESeq2 in larval motor neuron axons

Table S4. List of significantly differentially expressed genes identified by DESeq2 in motor neuron axons of adult legs

## References

[1] Rangaraju V., Tom Dieck S., Schuman E.M. (2017). Local translation in neuronal compartments: how local is local?. EMBO Rep..

[2] Bourke A.M., Schwarz A., Schuman E.M. (2023). De-centralizing the Central Dogma: mRNA translation in space and time. Mol. Cell.

[3] Holt C.E., Martin K.C., Schuman E.M. (2019). Local translation in neurons: visualization and function. Nat. Struct. Mol. Biol..

[4] Lin J.Q., van Tartwijk F.W., Holt C.E. (2021). Axonal mRNA translation in neurological disorders. RNA Biol..

[5] Heiman M., Kulicke R., Fenster R.J., Greengard P., Heintz N. (2014). Cell type-specific mRNA purification by translating ribosome affinity purification (TRAP). Nat. Protoc..

[6] Shigeoka T., Jung H., Jung J., Turner-Bridger B., Ohk J., Lin J.Q., Amieux P.S., Holt C.E. (2016). Dynamic Axonal Translation in Developing and Mature Visual Circuits. Cell.

[7] Ostroff L.E., Santini E., Sears R., Deane Z., Kanadia R.N., LeDoux J.E., Lhakhang T., Tsirigos A., Heguy A., Klann E. (2019). Axon TRAP reveals learning-associated alterations in cortical axonal mRNAs in the lateral amgydala. eLife.

[8] Garat J., Di Paolo A., Eastman G., Castillo P.E., Sotelo-Silveira J. (2025). The Trail of Axonal Protein Synthesis: Origins and Current Functional Landscapes. Neuroscience.

[9] Zhang K.X., Tan L., Pellegrini M., Zipursky S.L., McEwen J.M. (2016). Rapid Changes in the Translatome during the Conversion of Growth Cones to Synaptic Terminals. Cell Rep..

[10] Bertin B., Renaud Y., Aradhya R., Jagla K., Junion G. (2015). TRAP-rc, Translating Ribosome Affinity Purification from Rare Cell Populations of Drosophila Embryos. J. Vis. Exp..

[11] Thomas A., Lee P.J., Dalton J.E., Nomie K.J., Stoica L., Costa-Mattioli M., Chang P., Nuzhdin S., Arbeitman M.N., Dierick H.A. (2012). A versatile method for cell-specific profiling of translated mRNAs in Drosophila. PLoS One.

[12] Pallos J., Jeng S., McWeeney S., Martin I. (2021). Dopamine neuron-specific LRRK2 G2019S effects on gene expression revealed by translatome profiling. Neurobiol. Dis..

[13] Lehmkuhl E.M., Loganathan S., Alsop E., Blythe A.D., Kovalik T., Mortimore N.P., Barrameda D., Kueth C., Eck R.J., Siddegowda B.B. (2021). TDP-43 proteinopathy alters the ribosome association of multiple mRNAs including the glypican Dally-like protein (Dlp)/GPC6. Acta Neuropathol. Commun..

[14] Mahr A., Aberle H. (2006). The expression pattern of the Drosophila vesicular glutamate transporter: a marker protein for motoneurons and glutamatergic centers in the brain. Gene Expr. Patterns.

[15] Carrillo R.A., Özkan E., Menon K.P., Nagarkar-Jaiswal S., Lee P.T., Jeon M., Birnbaum M.E., Bellen H.J., Garcia K.C., Zinn K. (2015). Control of Synaptic Connectivity by a Network of Drosophila IgSF Cell Surface Proteins. Cell.

[16] Venkatasubramanian L., Guo Z., Xu S., Tan L., Xiao Q., Nagarkar-Jaiswal S., Mann R.S. (2019). Stereotyped terminal axon branching of leg motor neurons mediated by IgSF proteins DIP-α and Dpr10. eLife.

[17] Wang Y., Lobb-Rabe M., Ashley J., Chatterjee P., Anand V., Bellen H.J., Kanca O., Carrillo R.A. (2022). Systematic expression profiling of Dpr and DIP genes reveals cell surface codes in Drosophila larval motor and sensory neurons. Development.

[18] Taylor A.M., Berchtold N.C., Perreau V.M., Tu C.H., Li Jeon N., Cotman C.W. (2009). Axonal mRNA in uninjured and regenerating cortical mammalian axons. J. Neurosci..

[19] Shigeoka T., Koppers M., Wong H.H.W., Lin J.Q., Cagnetta R., Dwivedy A., de Freitas Nascimento J., van Tartwijk F.W., Ströhl F., Cioni J.M. (2019). On-Site Ribosome Remodeling by Locally Synthesized Ribosomal Proteins in Axons. Cell Rep..

[20] Farias J., Holt C.E., Sotelo J.R., Sotelo-Silveira J.R. (2020). Axon microdissection and transcriptome profiling reveals the in vivo RNA content of fully differentiated myelinated motor axons. RNA.

[21] Briese M., Saal L., Appenzeller S., Moradi M., Baluapuri A., Sendtner M. (2016). Whole transcriptome profiling reveals the RNA content of motor axons. Nucleic Acids Res..

[22] Nijssen J., Aguila J., Hoogstraaten R., Kee N., Hedlund E. (2018). Axon-Seq Decodes the Motor Axon Transcriptome and Its Modulation in Response to ALS. Stem Cell Rep..

[23] Roque C.G., Wong H.H.W., Lin J.Q., Holt C.E. (2016). Tumor protein Tctp regulates axon development in the embryonic visual system. Development.

[24] Gouveia Roque C., Holt C.E. (2018). Growth Cone Tctp Is Dynamically Regulated by Guidance Cues. Front. Mol. Neurosci..

[25] Piol D., Robberechts T., Da Cruz S. (2023). Lost in local translation: TDP-43 and FUS in axonal/neuromuscular junction maintenance and dysregulation in amyotrophic lateral sclerosis. Neuron.

[26] Moens T.G., Da Cruz S., Neumann M., Shelkovnikova T.A., Shneider N.A., Van Den Bosch L. (2025). Amyotrophic lateral sclerosis caused by FUS mutations: advances with broad implications. Lancet Neurol..

[27] Lindahl L. (2024). Ribosome Structural Changes Dynamically Affect Ribosome Function. Int. J. Mol. Sci..

[28] VanInsberghe M., van den Berg J., Andersson-Rolf A., Clevers H., van Oudenaarden A. (2021). Single-cell Ribo-seq reveals cell cycle-dependent translational pausing. Nature.

[29] Li H., Handsaker B., Wysoker A., Fennell T., Ruan J., Homer N., Marth G., Abecasis G., Durbin R., 1000 Genome Project Data Processing Subgroup (2009). The Sequence Alignment/Map format and SAMtools. Bioinformatics.

[30] Kim D., Paggi J.M., Park C., Bennett C., Salzberg S.L. (2019). Graph-based genome alignment and genotyping with HISAT2 and HISAT-genotype. Nat. Biotechnol..

[31] Love M.I., Huber W., Anders S. (2014). Moderated estimation of fold change and dispersion for RNA-seq data with DESeq2. Genome Biol..

[32] Ewels P., Magnusson M., Lundin S., Käller M. (2016). MultiQC: summarize analysis results for multiple tools and samples in a single report. Bioinformatics.

[33] Wang L., Wang S., Li W. (2012). RSeQC: quality control of RNA-seq experiments. Bioinformatics.

[34] Greene J.C., Whitworth A.J., Kuo I., Andrews L.A., Feany M.B., Pallanck L.J. (2003). Mitochondrial pathology and apoptotic muscle degeneration in Drosophila parkin mutants. Proc. Natl. Acad. Sci. USA.

[35] Parton R.M., Vallés A.M., Dobbie I.M., Davis I. (2010). Drosophila larval fillet preparation and imaging of neurons. Cold Spring Harb. Protoc..

[36] Brent J.R., Werner K.M., McCabe B.D. (2009). Drosophila larval NMJ dissection. J. Vis. Exp..

[37] Tahoe N.M.A., Mokhtarzadeh A., Curtsinger J.W. (2004). Age-related RNA decline in adult Drosophila melanogaster. J. Gerontol. A Biol. Sci. Med. Sci..

[38] Krueger F. Trim Galore!. https://www.bioinformatics.babraham.ac.uk/projects/trim_galore/.

[39] Andrews S. FASTQC. https://www.bioinformatics.babraham.ac.uk/projects/fastqc.

[40] Andrews S. Seqmonk. https://www.bioinformatics.babraham.ac.uk/projects/seqmonk/.

[41] Chen E.Y., Tan C.M., Kou Y., Duan Q., Wang Z., Meirelles G.V., Clark N.R., Ma'ayan A. (2013). Enrichr: interactive and collaborative HTML5 gene list enrichment analysis tool. BMC Bioinf..

[42] Pfaffl M.W. (2001). A new mathematical model for relative quantification in real-time RT-PCR. Nucleic Acids Res..

