# Supplementary material for: An optimized translating ribosome affinity purification protocol for low-abundance Drosophila tissues
Source: iScience. 2026 Mar 30;29(5):115530. doi: 10.1016/j.isci.2026.115530 (PMC13099348; doi:10.1016/j.isci.2026.115530)
Supplement: Document S1. Figures S1–S5 and Tables S1 and S5 [file mmc1.pdf]

iScience, Volume *29*

Supplemental information

An optimized translating ribosome affinity purification protocol for low-abundance *Drosophila* tissues

Leonor Miller-Fleming, Wing Hei Au, and Alexander J. Whitworth

Supplementary Information

Document S1. Figures S1–S5, Tables S1 and S5.

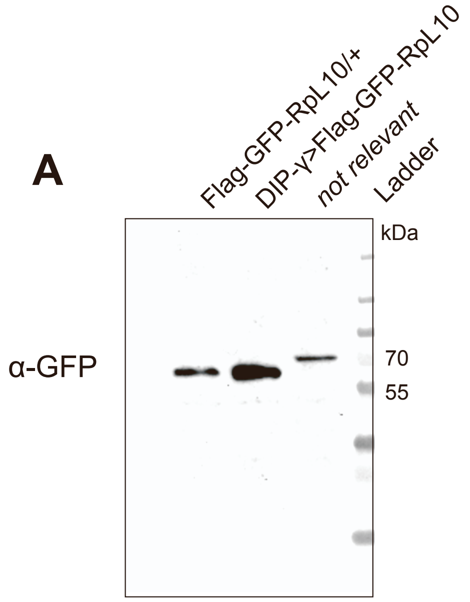

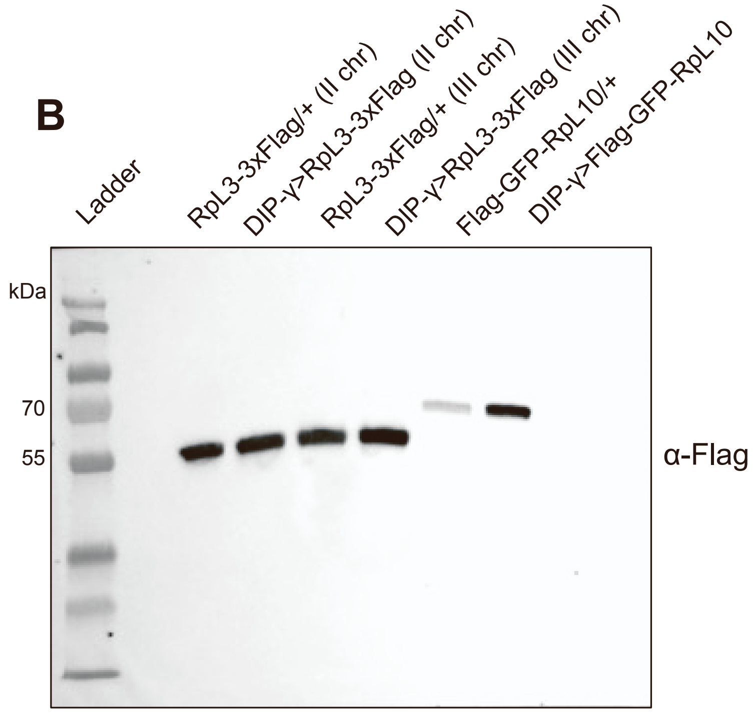

Figure S1. Leaky expression of UAS-Flag-GFP-RpL10. (A) Immunoblot analysis of Flag-GFP-RpL10/+ and DIP-γ>Flag-GFP-RpL10 fly lysates. (B) Immunoblot analysis of RpL3-3xFlag/+ and DIP-γ>RpL3-3xFlag fly lysates using two different insertions of RpL3-3xFlag on chromosome (Chr) II and III. Equivalent amounts of total proteins were loaded per sample. Lysates were enriched for DIP-γ neurons by removing fly abdomen before lysis.

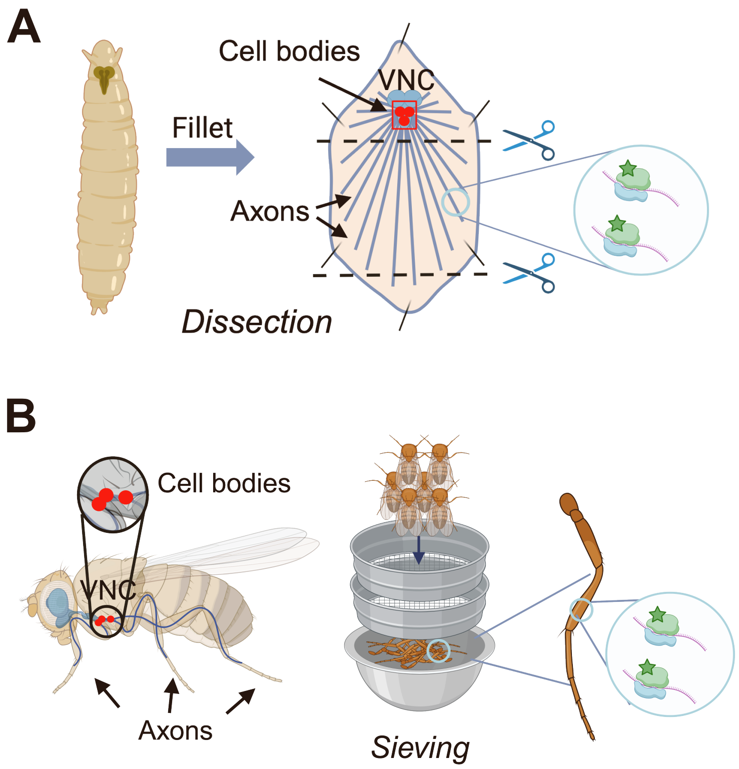

Figure S2. Isolation of MN axons from cell bodies in *Drosophila* larvae and adults. (A) Wandering L3 larvae are dissected as fillets, maintaining MNs intact. Both anterior (containing the VNC) and posterior ends are cut and discarded. The central portion of the fillet containing the MN axons is processed for TRAP. (B) Scheme representing the adult leg MN cell bodies localised in the VNC with axons projected into the leg, and isolation of adult legs using sieves. Adult legs are then processed for TRAP. VNC = ventral nerve cord.

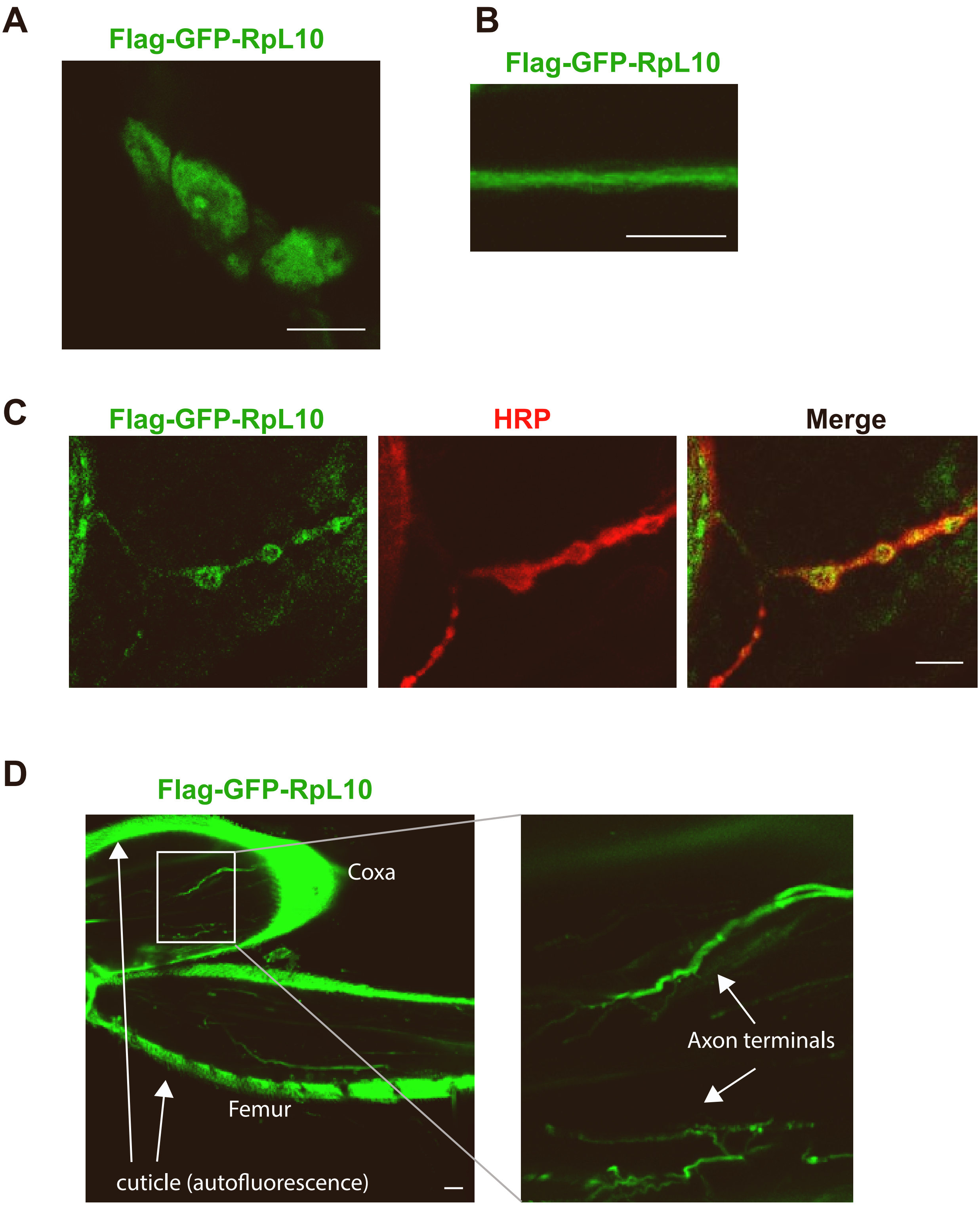

Figure S3. Expression of Flag-GFP-RpL10 in larval and adult leg motor neurons. (A-C) Flag-GFP-RpL10 expression driven by OK371-GAL4 in larval motor neuron cell bodies (A), axons (B), and neuromuscular junctions (NMJs) (C). NMJs were co-stained with HRP and Alexa Fluor 568. (D) Flag-GFP-RpL10 expression in adult leg motor neurons driven by DIP-γ-GAL4. The inset shows a higher-magnification view of the axon terminals. Scale bars: 10 μm (A-C); 20 μm (D).

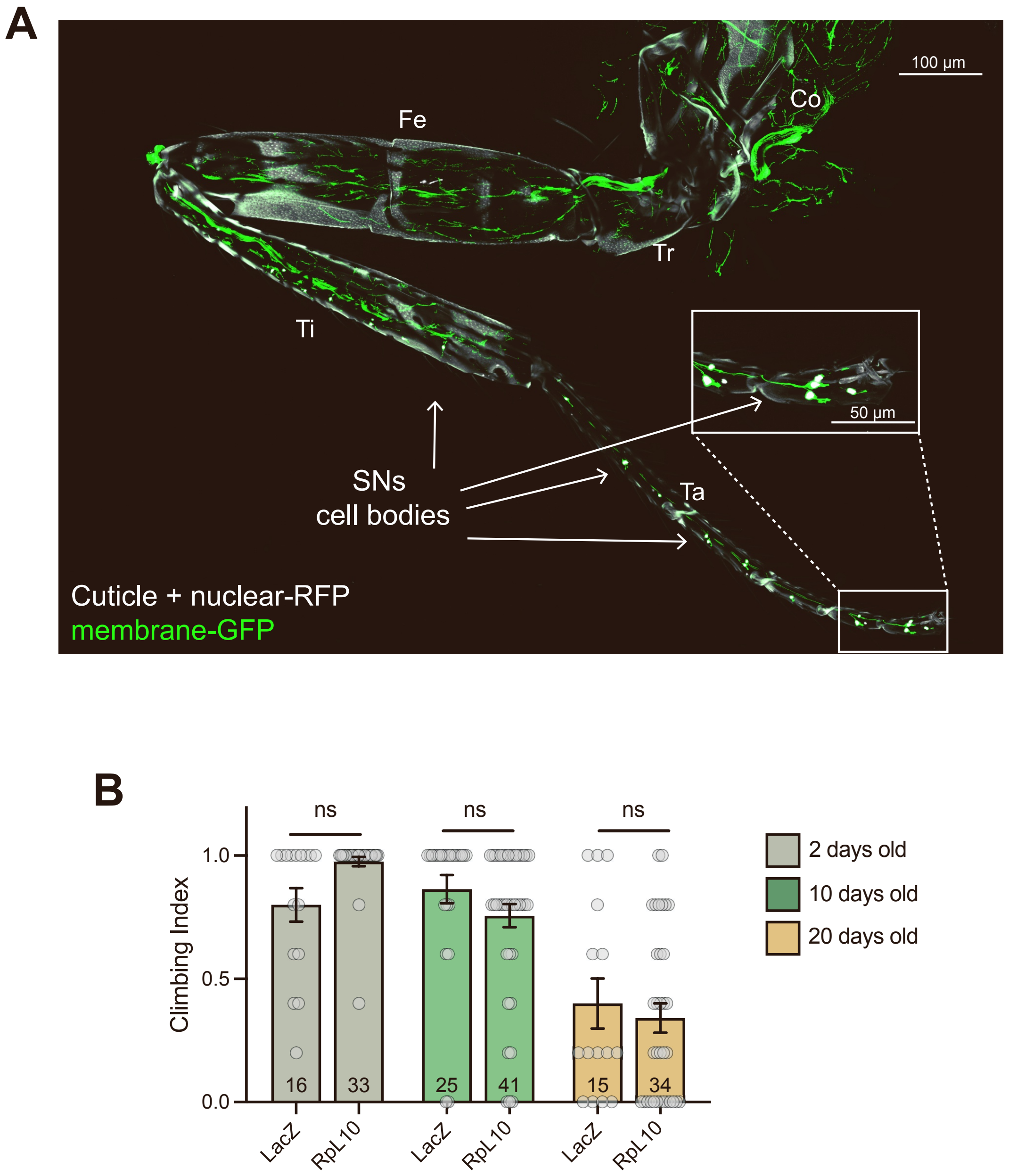

Figure S4. Expression pattern of OK371-GAL4 in the adult leg. (A) OK371-GAL4 was used to drive expression of membrane-GFP, labelling axons, and nuclear-RFP, to highlight cell bodies. Some autofluorescence of cuticle is detected in the RFP channel. Although mainly considered a MN driver, OK371-GAL4 also expresses in a few sensory nerves (SNs) in the leg. Co = coxa, Tr = trochanter, Fe = femur, Ti = tibia, Ta = tarsus. (B) Climbing assay of DIP-γ-GAL4 driven Flag-GFP-RpL10 or LacZ control at the indicated ages. Chart shows mean ± SEM. Number of animals (N) is shown in the bars. ns = not significant.

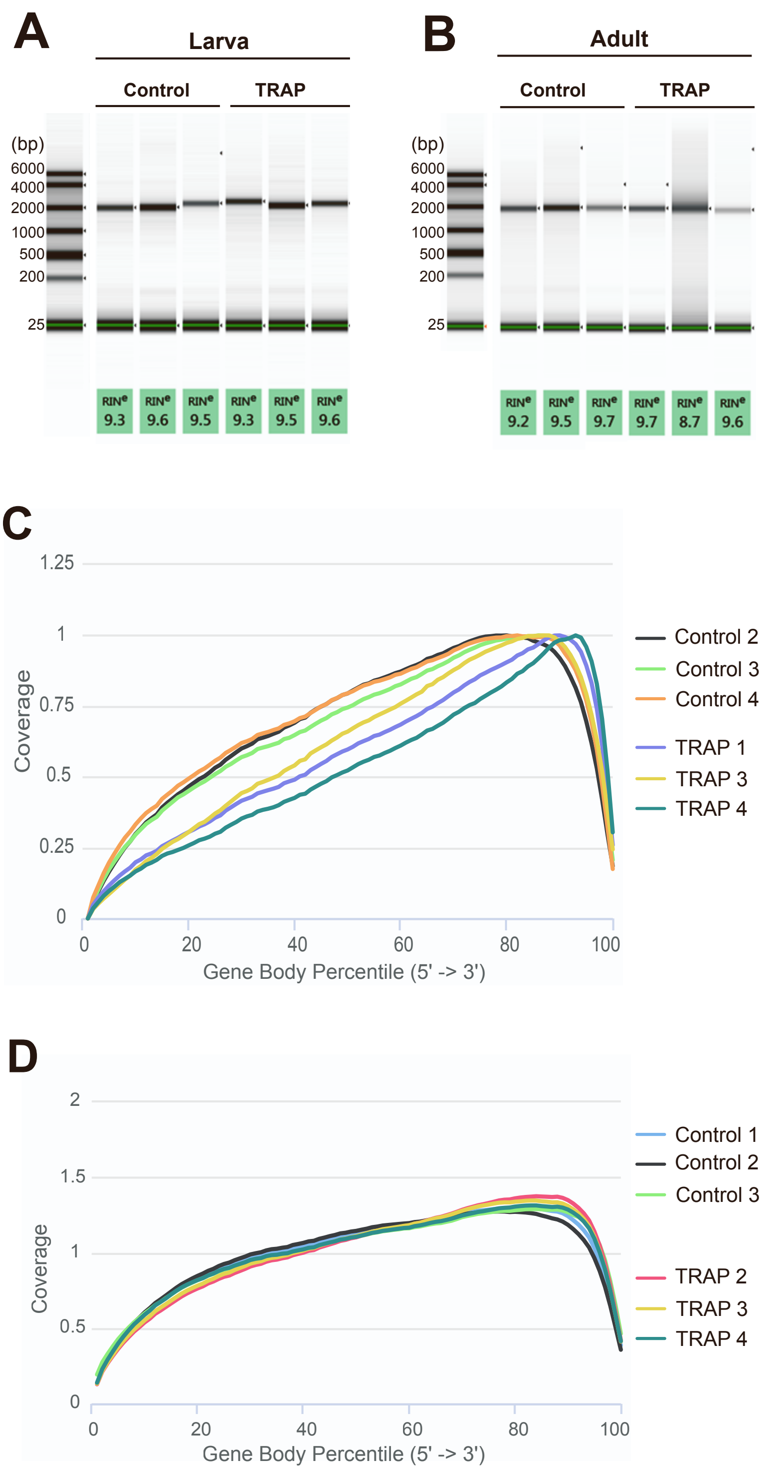

Figure S5. RNA quality of MN-axon TRAP samples. TapeStation electrophoresis of RNAs eluted from axonal TRAPs using (A) L3 larvae OK371>Flag-GFP-RpL10 axonal fractions and (B) adult DIP-γ>Flag-GFP-RpL10 legs, compared to matched controls (Flag-GFP-RpL10/+). Gene body coverage across normalised transcript lengths assessed in larval (C) and adult leg (D) samples.

Parameters	Optimisation step tested	Outcome/Technical limitation identified	Final step implemented
Antibody/bead conjugates	Tested multiple antibody/beads conjugates	Some antibody/beads conjugates immunoprecipitated RpL10 efficiently, but caused high unspecific RNA background	Selected Dynabeads + anti-Flag (Monoclonal mouse anti-FLAG M2 antibody, F1804), which showed the lowest RNA background
Buffer:tissue ratio	Tested multiple ratios	RNA degradation was observed if ratio of buffer:tissue was low	Selected the appropriate ratio to prevent degradation
Tissue lysis	Evaluated different lysis buffers, with and without detergent	Inclusion of detergent during lysis increased RNA background, particularly in larval samples	Detergent was added only after lysis and centrifugation to minimise background
RNase inhibitors	Different RNase inhibitors	When RNA degradation was observed, no differences were detected between different RNase inhibitors	RNA degradation was reduced through protocol adjustments (strict cold handling, rapid processing), rather than inhibitor selection.
Pre-clearing lysate	Tested multiple pre-clearing duration	Longer pre-clearing times showed reduced RNA background, but increased RNA degradation	Pre-clearing step was included, but with a short incubation time
Immuno-precipitation time	Tested multiple incubation times	Longer immunoprecipitation improved ribosome recovery but also increased RNA degradation	2 hours incubation was the optimal balance between ribosome yield and RNA integrity

Table S1. Summary of TRAP protocol steps that were optimised for this protocol.

Target gene	**Oligo sequence**	
GFP	Forward	GACAACCACTACCTGAGCAC
	Reverse	CAGGACCATGTGATCGCG
18S	Forward	GACACGCAAACTTCTCAACAG
	Reverse	CTTCAGAGCCAATCCTTATCCC
28S	Forward	AAACGGCTACCACATCTAAGG
	Reverse	ATTCCAATTACAGGGCCTCG
VGlut1	Forward	CGTCTGGGAAAATCAGGACTAC
	Reverse	CACCATGATAGCTCTGCGAT
Futsch	Forward	CGAGGAGCACAAGGACAATATC
	Reverse	CAGGTGCATGCTGTGTAATTG
Act42A	Forward	GCGTCGGTCAATTCAATCTT
	Reverse	AAGCTGCAACCTCTTCGTCA
Mef2	Forward	AAGTGATCCACGCGAGTTC
	Reverse	TTCCGTATAAATTGTCCCCAGG
mt:ND3	Forward	AAAAAGCTTTAATCGACCGAGA
	Reverse	CGTAAAGAAAATGGTAATCGAGATG
Acp1	Forward	GATCCTTTCATACCTGTCCCG
	Reverse	GAGAAATAGGCAACCACAAACG

Table S5. List of primers used.
